# Oncogenic KRAS Rewires Stress Granule Dynamics: Mechanisms and Therapeutic Opportunities

**DOI:** 10.1002/kjm2.70196

**Published:** 2026-03-02

**Authors:** Msimisi Ndzinisa, Birhanetensay Masresha Altaye, Yuh‐Pyng Sher

**Affiliations:** ^1^ Graduate Institute of Biological Science and Technology China Medical University Taichung Taiwan; ^2^ Graduate Institute of Biomedical Sciences China Medical University Taichung Taiwan; ^3^ Institute of Biochemistry and Molecular Biology China Medical University Taichung Taiwan; ^4^ Cancer Biology and Precision Therapeutics Center China Medical University Taichung Taiwan; ^5^ Center for Molecular Medicine China Medical University Hospital Taichung Taiwan

**Keywords:** cancer therapy resistance, KRAS mutation, RNA‐binding proteins, stress granules, targeted therapies

## Abstract

Stress granules (SGs) are dynamic, membrane‐less structures that form in response to various cellular stresses, including metabolic, oxidative, and therapeutic challenges. They function as adaptive hubs and reorganize protein synthesis and signaling networks to help cells survive under stress. In cancer, these condensates are often hijacked to support survival and therapy resistance. SGs can sequester proapoptotic factors, buffer metabolic‐ and treatment‐induced stress, and stabilize transcripts that promote cell survival, collectively contributing to tumor aggressiveness and resistance to therapy. Their formation relies on protein–RNA interactions, phase separation, and posttranslational modifications, which tumor cells exploit to maintain SGs even when normal stress conditions trigger their disassembly. This creates a protective pool of mRNAs and proteins that allows rapid adaptation to stress. Emerging therapeutic strategies that disrupt SG assembly, interfere with the adaptive functions of SGs, or accelerate SG clearance have shown promise in sensitizing tumors to treatment. This review summarizes the current understanding of SG dynamics, illustrating how cancer cells exploit these structures to survive stress. We focus specifically on KRAS‐driven cancers, where persistent oncogenic signaling enhances SG formation and stability, making these condensates critical mediators of tumor adaptation. Targeting SG formation, maintenance, or associated stress‐response pathways represents a promising approach to enhance therapeutic efficacy and improve long‐term outcomes in cancers driven by sustained cellular stress.

**Abbreviations**
15‐d‐PGJ_2_ (15d‐PGJ_2_): 15‐deoxy‐Δ^12,14^‐prostaglandin J_2_
4E‐BP1: eukaryotic translation initiation factor 4E‐binding protein 1
AAA+: ATPases associated with diverse cellular activities
ADMA: asymmetric dimethylarginine
APC: adenomatous polyposis coli
ATF4: activating transcription factor 4
ATP: adenosine triphosphate
BCR–ABL: breakpoint cluster region–Abelson fusion oncoprotein
CALCOCO2 (NDP52): calcium binding and coiled‐coil domain 2
CB‐839: glutaminase inhibitor
COX‐2: cyclooxygenase‐2
DDX3X: DEAD‐box helicase 3 X‐linked
DDX6: DEAD‐box helicase 6
DHX36: DEAH‐box helicase 36
eIF2: eukaryotic initiation factor 2
eIF2B: eukaryotic initiation factor 2B
eIF2α: eukaryotic initiation factor 2 alpha subunit
eIF4A: eukaryotic initiation factor 4A
eIF4E: eukaryotic initiation factor 4E
eIF4GI: eukaryotic initiation factor 4 gamma 1
ER: endoplasmic reticulum
ERK: extracellular signal‐regulated kinase
FUS: fused in sarcoma
G3BP1: Ras‐GTPase‐activating protein SH3‐domain‐binding protein 1
G3BP2: Ras‐GTPase‐activating protein SH3‐domain‐binding protein 2
G3Ia/G3Ib: G3BP inhibitors A and B
GADD34: growth arrest and DNA damage‐inducible protein 34
GCN2: general control nonderepressible 2
GDP: guanosine diphosphate
GTP: guanosine triphosphate
HDAC: histone deacetylase
HDAC6: histone deacetylase 6
HPGD: 15‐hydroxyprostaglandin dehydrogenase
HRI: heme‐regulated inhibitor
HSP70: heat shock protein 70
HSPB8: heat shock protein family B (small) member 8
IDR: intrinsically disordered region
IGF1: insulin‐like growth factor 1
ISR: integrated stress response
ISRIB: integrated stress response inhibitor
KRAS: Kirsten rat sarcoma viral oncogene homolog
KRASG12C/KRASG12D: KRAS glycine‐to‐cysteine/aspartate mutation at codon 12
LLPS: liquid–liquid phase separation
LSL: Lox–Stop–Lox
m^6^A: N6‐methyladenosine
MAPK: mitogen‐activated protein kinase
MEK: MAPK/ERK kinase
MG53 (TRIM72): mitsugumin 53
mRNA: messenger RNA
mRNP: messenger ribonucleoprotein
MSGP: mammalian stress granule proteome
mTOR: mechanistic target of rapamycin
mTORC1: mTOR complex 1
NEDDylation: neural precursor cell expressed developmentally downregulated protein 8 conjugation
NRF2: nuclear factor erythroid 2‐related factor 2
NSCLC: non‐small cell lung cancer
nsP3: nonstructural protein 3
NTF2L: nuclear transport factor 2‐like domain
NUPR1: nuclear protein 1
O‐GlcNAc: O‐linked *N*‐acetylglucosamine
OGT: O‐linked *N*‐acetylglucosamine transferase
PABP1 (PABPC1): poly(A)‐binding protein 1
PARP13: poly(ADP‐ribose) polymerase family member 13
PARylation: poly(ADP‐ribosyl)ation
PDAC: pancreatic ductal adenocarcinoma
PD‐L1: programmed death‐ligand 1
PERK: PKR‐like endoplasmic reticulum kinase
PI3K: phosphoinositide 3‐kinase
PKR: protein kinase R
PP1: protein phosphatase 1
PRMT1: protein arginine methyltransferase 1
PTM: posttranslational modification
RAF: rapidly accelerated fibrosarcoma kinase
RBP: RNA‐binding protein
RNF144A: ring finger protein 144A
RNP: ribonucleoprotein
ROS: reactive oxygen species
SG: stress granule
SOD1: superoxide dismutase 1
SQSTM1 (p62): sequestosome 1
SRPK2: serine/arginine protein‐specific kinase 2
SRSF3: serine/arginine‐rich splicing factor 3
SUMOylation: small ubiquitin‐like modifier conjugation
TDRD3: Tudor domain‐containing protein 3
TIA‐1: T‐cell‐restricted intracellular antigen‐1
TIAR: TIA‐1‐related protein
TRIM37: tripartite motif‐containing protein 37
tRNAᵢᴹᵉᵗ: initiator methionyl transfer RNA
TTP: tristetraprolin
UBAP2L: ubiquitin‐associated protein 2‐like
UDP‐GlcNAc: uridine diphosphate *N*‐acetylglucosamine
UV: ultraviolet
VCP (p97): valosin‐containing protein
VRK2: vaccinia‐related kinase 2
YAP: Yes‐associated protein
YTHDF: YTH domain family protein
Zn^2+^
: zinc ion

## 1 Introduction

Stress granules (SGs) represent transient, dynamic, membrane‐less ribonucleoprotein (RNP) condensates that are orchestrated in response to diverse physiological insults, such as oxidative, endoplasmic reticulum (ER), metabolic, and replicative stresses [1]. Morphologically, SGs appear as short‐lived cytoplasmic foci with dimensions ranging from 100 to 2000 nm that are detectable by light microscopy. First described in 1986, SGs are now widely recognized as adaptive assemblies that protect cells by sequestering untranslated mRNAs with RNA‐binding proteins (RBPs), thereby alleviating the burden on the protein synthesis machinery and conserving energy to promote survival under adverse conditions [2].

Liquid–liquid phase separation (LLPS) drives SG assembly, facilitating the condensation of proteins and RNAs into dynamic, liquid‐like droplets [3]. Two proposed models describe this assembly: the core‐first model, in which the initial formation of untranslated dense scaffolds of mRNA and RNPs (mRNPs) recruits the surrounding shell through weaker interactions, and the LLPS‐first model, in which multivalent interactions at high mRNP concentrations trigger phase separation prior to the maturation of internal core structures. In nonmalignant cells, SGs are characterized by their fluidity and reversibility, sequestering the transcriptome during acute stress and undergoing dissolution upon the restoration of cellular homeostasis. In addition to their typical role, SGs serve an essential atypical function as rapid responders to endomembrane damage. By acting as a physical “molecular plug,” SGs provide an immediate scaffold for the stabilization of the lysosomal membrane within seconds of injury, establishing SGs as critical mediators of membrane integrity and cellular survival following organelle‐specific stress [4]. On the other hand, in malignant cells, SGs often persist aberrantly and adopt a solid‐like state that renders them resistant to autophagic clearance and therapy. This process is frequently driven by mutations in RBP genes, such as *FUS* [5] or the DEAD‐box helicase *DDX3X* [6], which disrupt SG dynamics. Furthermore, the sequestration of aggregation‐prone proteins, including mutant SOD1, promotes a transition toward solid‐like states [7].

Persistent stress stimuli collectively trigger eukaryotic initiation factor 2α (eIF2α) phosphorylation, a process that limits the availability of the eIF2–GTP–tRNAᵢᴹᵉᵗ ternary complex, consisting of eukaryotic initiation factor 2 (eIF2), GTP, and initiator methionyl‐tRNA (tRNAᵢᴹᵉᵗ), thereby suppressing canonical translation and initiating SG assembly. By selectively sequestering mRNAs associated with tumor suppression and apoptosis within SGs [8, 9], this process enables the preferential synthesis of cytoprotective proteins, thereby achieving adaptive fitness under adverse conditions in cancer cells. Although SGs are characterized primarily as sites of translational silencing, activating transcription factor 4 (ATF4), which is preferentially translated in response to eIF2α phosphorylation, serves as a major regulator of the complete translation cycle of transcripts localized to SGs [10]. These findings suggest that SGs allow select transcripts to reenter the active translation pool.

SGs are composed of distinct functional constituents, including nucleators, which are the primary drivers of LLPS, and mediators, which recruit specific RNA and protein cargo. Ras‐GTPase‐activating protein SH3‐domain‐binding proteins (G3BPs), particularly G3BP1 and G3BP2, serve as essential nucleators and markers of SGs [11]. Although G3BP1 drives condensation, it frequently localizes to the shell rather than the core, where it organizes auxiliary SG components [1]. Furthermore, SGs sequester nontranslating mRNAs and 40S ribosomal subunits; these act as core scaffold elements that facilitate translational arrest.

SG dynamics are regulated by oncogenic and stress‐response pathways altered by gene mutations. Mutant p53 suppresses SG formation through interactions with PERK and G3BP1, creating therapeutic vulnerability in cancer cells [12]. YAP preserves the fluidity of biomolecular condensates and prevents their pathological aggregation [13]. The BCR–ABL fusion oncoprotein is localized to SGs, where it supports oncogenic signaling and contributes to chronic myeloid leukemia pathogenesis [14]. Among the oncogenic drivers that exploit the SG machinery, KRAS mutations play a prominent role. Accordingly, this review focuses on the KRAS‐mediated regulation of SG formation, given the high prevalence of KRAS mutations in pancreatic cancer (90%–95%) [15], colorectal cancer (40%–50%) [16], and lung adenocarcinoma (25%–30%) [17]. These mutations impose persistent intrinsic cellular stress from the earliest stages of tumorigenesis [18]. Consistently, increased SG assembly and defective SG disassembly have emerged as key downstream consequences of oncogenic KRAS signaling, suggesting that SGs are potentially vulnerable in KRAS‐mutant cancers.

## 2 Stress Granule Dynamics and Function

### 2.1 SG Initiation

Cellular stress in cancer cells triggers the formation of SGs, which are dynamic ribonucleoprotein condensates that protect cells under adverse conditions. In response to diverse stress stimuli, the integrated stress response (ISR) is activated through four principal stress‐sensing kinases: PKR‐like endoplasmic reticulum kinase (PERK), general control nonderepressible 2 (GCN2), heme‐regulated inhibitor (HRI), and protein kinase R (PKR) [19]. These ISR kinases can phosphorylate eIF2α at Ser51. When eIF2α is phosphorylated, it sequesters and inhibits its guanine nucleotide exchange factor eIF2B, preventing the recycling of eIF2–GDP to eIF2–GTP. This depletes the eIF2–GTP–tRNAᵢᴹᵉᵗ ternary complex, thereby stalling translation initiation and causing mRNAs to accumulate in incomplete 48S preinitiation complexes.

Mutant KRAS induces hyperactive signaling that drives excessive metabolic and proliferative demand, resulting in multiple cellular stresses, including metabolic, proteotoxic, genotoxic, and oxidative microenvironments, which converge on translational inhibition via canonical (eIF2α phosphorylation) or noncanonical (e.g., 15‐d‐PGJ_2_‐mediated eIF4A inhibition) pathways, generating stalled mRNPs that nucleate stress granules and increase tumor survival and chemoresistance. Releasing from polysomes, long single‐stranded unfolded RNAs can induce conformational changes in the original compact autoinhibited state of G3BP1 and other nucleators, thereby enhancing local multivalent interactions that initiate stress granule formation [20].

SGs can also be initiated by chemotherapy in a p‐eIF2α‐dependent manner [21]. Blocking p‐eIF2α using a dominant‐negative eIF2α mutation reduces SG formation and sensitizes cells to chemotherapy [22]. The size, number, and dynamics of SGs vary with the drug: 5‐fluorouracil and bortezomib strongly promote G3BP1‐dependent SG condensation, whereas cisplatin typically induces smaller or more transient SGs [21]. These SGs collectively increase tumor cell survival and confer chemoresistance, depending on the cell type and stress context. In contrast to those with p‐eIF2α‐dependent translation inhibition, colorectal cancers with *APC* loss maintain high levels of p‐eIF2α. However, the eIF2B complex recognizes p‐eIF2α to selectively translate growth‐promoting mRNAs, thereby supporting tumor survival through an alternative regulatory pathway [23].

### 2.2 Stress Granule Formation

SGs form through LLPS, which is driven by multivalent interactions, including protein–protein interactions such as G3BP1 dimerization, protein–RNA binding, and RNA–RNA interactions [24]. Epitranscriptomic modifications such as m6A further bias transcript recruitment into SGs by engaging LLPS‐competent m6A reader proteins (e.g., YTHDF family members), thereby reinforcing RNA‐driven condensation [25]. The stress‐response gene nuclear protein 1 (NUPR1) similarly contributes to SG formation by promoting LLPS and reinforcing granule stability [26]. G3BP1 upregulation alters SG dynamics and promotes tumor growth in KRAS‐mutant cancers, underscoring its role in oncogenic stress adaptation. As a central nucleator of SG formation, G3BP1 facilitates multivalent interactions through its C‐terminal α‐helical RNA‐binding domain and scaffolds additional nucleating factors, including PABP1, TIA‐1, caprin‐1, and TIAR [27]. A key feature of G3BP1 is its intrinsically disordered region (IDR), a segment lacking a stable three‐dimensional structure. While structured domains confer binding specificity, the IDRs in most SG components provide the conformational flexibility and multivalency required to simultaneously bridge diverse RNA molecules and protein partners. Proteins such as TIA‐1 further facilitate LLPS via their prion‐like domains, self‐association, RNA recognition motifs, and Zn^2+^‐stabilized contacts, forming dynamic, reversible condensates that nucleate SGs and recruit other RNA‐binding proteins [28]. The kinetics and dynamics of these interactions are finely tuned by helicases such as DHX36, which unwind RNA structures that drive intermolecular RNA–RNA interactions, and by DDX6, which remodels RNPs to maintain the liquid‐like fluidity of SGs [29].

Recent studies have highlighted the critical role of posttranslational modifications (PTMs) in controlling stress granule assembly and stability. Protein arginine methyltransferase 1 (PRMT1) catalyzes asymmetric dimethylation of arginine residues (ADMA) on core SG proteins such as G3BP1 [30]. These modifications are recognized by Tudor domain‐containing protein 3 (TDRD3), which connects methylated G3BP1 to RNAs, promoting LLPS and granule formation. Interestingly, arginine demethylation of G3BP1 at Arg435, Arg447, and Arg460 has also been shown to enhance SG assembly [31]. Histone deacetylases (HDACs), particularly HDAC6, also facilitate stress granule formation by deacetylating G3BP1 at lysine‐376, which enhances its RNA‐binding and nucleation activity [32].

Another core nucleator, UBAP2L, is regulated by PRMT1‐mediated arginine methylation, which fine‐tunes its interactions with other stress granule components [33]. When PRMT1 activity is low, the methylation of UBAP2L is reduced, allowing it to engage more efficiently in multivalent protein–RNA interactions and thereby promoting the nucleation and assembly of stress granules. In addition, O‐GlcNAcylation of UBAP2L protects it from TRIM37‐mediated ubiquitination and proteasomal degradation, stabilizing UBAP2L protein levels and promoting stress granule assembly under cellular stress [34]. In KRAS‐mutant cancers, hyperactive glucose metabolism channels are involved in the hexosamine biosynthetic pathway, increasing UDP‐GlcNAc production and protein O‐GlcNAcylation, which supports tumor growth and stress adaptation [35]. Although direct experimental evidence is lacking, it is plausible that this enhanced O‐GlcNAcylation could modify RNA‐binding proteins or stress granule nucleators, thereby reinforcing stress granule assembly under oncogenic or chemotherapeutic stress [34]. Other PTMs also regulate stress granule dynamics, including PARylation, which modulates stress granule phase separation and maturation through poly (ADP‐ribose)‐dependent interactions with RNA‐binding proteins [36], such as PARP13, and SUMOylation of PABPC1, which stabilizes U‐rich mRNAs within stress granules to promote cell survival under stress [37]. In addition, stress‐induced NEDDylation of the SG assembly factor serine/arginine‐rich splicing factor 3 (SRSF3) at Lys85 promotes efficient stress granule assembly by facilitating interactions with core SG components [38]. Together, these modifications demonstrate how SG components, coordinated by nucleators, intrinsically disordered regions, helicases, and PTMs, drive persistent stress granule formation and support tumorigenesis in KRAS‐driven cancers.

### 2.3 Stress Granule Degradation

While SG formation supports cancer cell survival under stress, KRAS‐driven signaling promotes persistent SGs that resist timely disassembly, creating a protective reservoir of mRNAs and proteins. Recovery from stress requires the dephosphorylation of eIF2α, which is mediated by the GADD34‐PP1 phosphatase complex [39, 40]. GADD34, a stress‐induced regulatory subunit, recruits the catalytic phosphatase PP1 to p‐eIF2α to facilitate dephosphorylation and restore translational activity. When translation resumes, mRNAs are recruited into polysomes, effectively depleting SG maintenance and thereby promoting granule disassembly. Pharmacological restoration of translation, such as with ISRIB, similarly accelerates SG clearance by overcoming p‐eIF2α‐dependent translational repression [41].

SG disassembly is a dynamic, multistep process involving the remodeling of RNA–RNA and RNA–protein interactions that ultimately results in the dissociation of SG cores and shells upon stress relief [42]. RNA helicases, including eIF4A, use ATP to unwind RNA and reorganize RNA–protein complexes. By disrupting the interactions that hold RNAs together in stress granules, they reduce granule stability and promote disassembly. In terms of complementing helicase activity, chaperone networks, including those of the HSP70/HSPB8 family, assist in the remodeling of misfolded or aggregated proteins, enhancing SG fluidity and contributing to clearance. The AAA+ ATPase Valosin‐containing protein (VCP/p97) promotes granule breakdown, and inhibition of VCP delays SG clearance following heat or oxidative stress in mammalian cells, underscoring its reliance on ATP‐driven protein remodeling to disassemble stable complexes [43].

The efficiency of these clearance pathways is regulated by PTMs. For instance, impaired SUMOylation of SG proteins and attenuated disassembly‐engaged proteins disrupt SG dissolution, which is linked to the pathogenesis of amyotrophic lateral sclerosis [44]. In KRAS‐mutant cells, metabolic alterations may further influence these PTM networks; specifically, fluctuations in acetyl‐CoA availability can alter the acetylation of SG components to promote granule persistence. SGs that evade immediate disassembly are subsequently targeted for selective autophagy (granulophagy), wherein receptors such as SQSTM1/p62 and CALCOCO2/NDP52 recognize SG cargo for lysosomal degradation. The relative contributions of helicases, chaperones, ubiquitin/SUMO modifications, VCP extraction, and autophagy vary with the type, duration, and intensity of stress, indicating that SG clearance is a highly context‐dependent process rather than a single, unified pathway [42].

Notably, small molecules can also modulate SG formation and clearance kinetics. Resveratrol and its derivatives have been shown to induce the formation of small stress granules by directly binding the core nucleator G3BP1, and these SGs are rapidly cleared upon stress removal [45]. These findings suggest that altering the protein–protein interactions of G3BP1 during SG assembly can influence clearance kinetics, providing a potential strategy for pharmacologically targeting SGs in cancer or stress contexts.

## 3 KRAS Signaling Promotes SG Assembly

**FIGURE 1:**
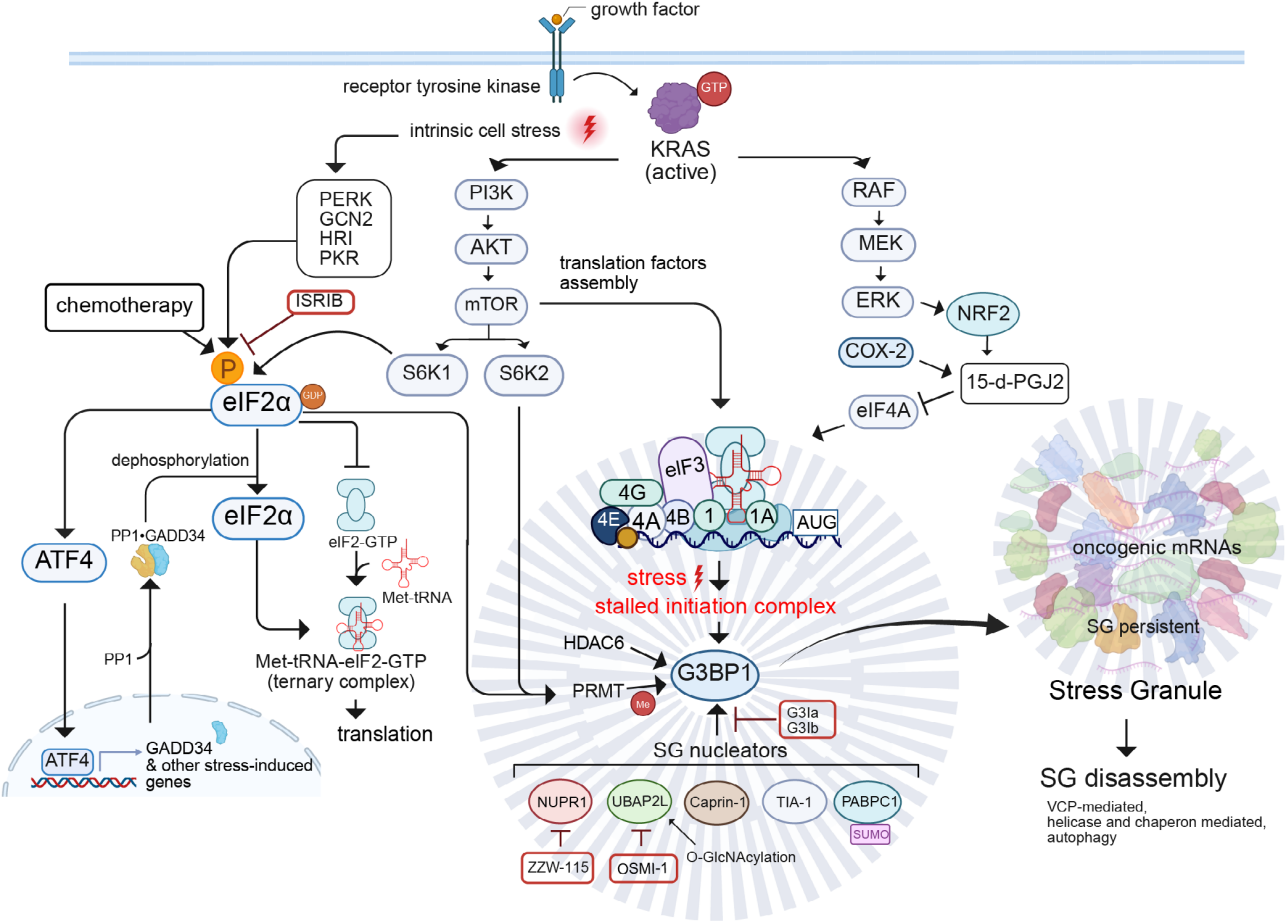
Mechanisms of KRAS‐mediated stress granule (SG) assembly under intrinsic and extrinsic stress. Intrinsic stress and chemotherapeutic agents trigger the integrated stress response by phosphorylating eIF2α via stress kinases (PERK, GCN2, HRI, and PKR). ISRIB serves as a small‐molecule inhibitor of this signaling axis. Phosphorylated eIF2α (P‐eIF2α) attenuates global translation by preventing ternary complex formation (Met‐tRNA–eIF2–GTP), resulting in the formation of stalled initiation complexes. Concurrently, selective ATF4 translation induces the expression of stress‐responsive genes, such as GADD34, for feedback regulation. Oncogenic KRAS modulates SG nucleation through divergent signaling axes. PI3K–AKT–mTOR signaling contributes to translation, but under stress, the assembled factors and translational components become stalled and nucleate SGs. The mTOR kinase S6K1 contributes to SGs via P‐eIF2α, and S6K2 stabilizes SGs. The RAF–MEK–ERK pathway elevates 15‐d‐PGJ2 levels through the induction of COX‐2 and NRF2 expression. 15‐d‐PGJ2 directly inhibits eIF4A, further promoting the accumulation of stalled complexes. These complexes are sequestered by the primary nucleator G3BP1 and associated factors (NUPR1, UBAP2L, Caprin‐1, TIA‐1, and PABPC1). SG stability is further regulated by posttranslational modifications, including methylation, SUMOylation, and O‐GlcNAcylation. Persistent SGs serve as reservoirs for oncogenic mRNAs, maintaining their stability until the granules undergo disassembly via VCP‐dependent extraction, RNA helicase activity, chaperone‐mediated refolding, or autophagic clearance.

Hyperactive KRAS signaling is involved in multiple downstream pathways that are involved in the regulation of SG assembly (Figure 1). mTOR effector S6 kinases (S6K1 and S6K2) localize to SGs and influence their dynamics in human cells [46]. S6K1 facilitates SG assembly by regulating eIF2α phosphorylation, linking mTOR signaling to canonical stress responses, whereas S6K2 promotes SG persistence. Further supporting this, the oncogenic mTORC1‐eIF4E pathway promotes SG formation through phosphorylation of 4E‐BP1, enabling eIF4E‐eIF4GI interactions. These interactions facilitate the assembly of cap‐dependent translation initiation complexes, which, upon stress‐induced inhibition, stall and serve as nucleation sites for SGs [46]. KRAS‐dependent mTORC1 activation likely converges on this pathway, promoting the recruitment of SG nucleators such as TIA1/TIAR and G3BP1, which further stabilize granules during stress. Disruption of this pathway could impair SG assembly and potentially sensitize cancer cells to apoptosis.

Consistent with these mechanisms, activation of the IGF1–PI3K–mTOR–S6K1 axis promotes SG assembly in obesity‐associated pancreatic ductal adenocarcinoma [47]. Specifically, mTORC1 activation leads to S6K1‐mediated phosphorylation of serine/arginine protein‐specific kinase 2 (SRPK2), which is required for SRPK2 localization to SGs. Within granules, SRPK2 acts as a structural scaffold, stabilizing the ribonucleoprotein network and promoting nucleation. Loss of SRPK2 or inhibition of its phosphorylation impairs SG formation, linking metabolic signaling via S6K1 directly to granule assembly [47]. These SGs further integrate KRAS‐driven prosurvival and chemoresistance pathways, highlighting the multifaceted contribution of KRAS to granule dynamics.

Oncogenic KRAS signaling increases chronic metabolic, oxidative, and proteotoxic stress while simultaneously activating antioxidant activity and survival pathways, leading to partial translational inhibition and the accumulation of untranslated mRNAs and stress granule nucleators. Mechanistically, KRAS activates nuclear factor erythroid 2‐related factor 2 (NRF2), a master transcriptional regulator of antioxidant and redox homeostasis, through the MAPK/ERK pathway. This activity enhances antioxidant capacity and maintains basal reactive oxygen species (ROS) levels at sublethal levels [48]. Redox buffering prevents oxidative stress‐induced apoptosis, allowing KRAS‐driven stress to persist without killing cells. While NRF2 does not directly nucleate SGs, this redox buffering prevents oxidative stress‐induced apoptosis and is required for efficient 15‐deoxy‐Δ^12,14^‐prostaglandin J_2_ (15‐d‐PGJ_2_)‐induced SG formation [49]. Moreover, KRAS intrinsically generates metabolic and proteotoxic stress through its oncogenic activity, which affects the ISR via PERK‐ and GCN2‐mediated phosphorylation of eIF2α [18]. Phosphorylation of eIF2α suppresses translation initiation and drives polysome collapse, leading to the accumulation of untranslated mRNAs within stalled 48S preinitiation complexes. These complexes are subsequently bound by core stress granule nucleators, including G3BP1, creating a cellular state that favors rapid stress granule assembly upon stress [50]. In addition to promoting mRNA accumulation, oncogenic RAS signaling actively suppresses mRNA decay pathways. Coelho et al. demonstrated that RAS–MEK–ERK signaling stabilizes PD‐L1 mRNA by inhibiting the RNA‐destabilizing protein tristetraprolin (TTP), thereby prolonging transcript half‐life independently of transcriptional regulation. This KRAS‐driven inhibition of mRNA decay likely creates a permissive environment for the sequestration and protection of untranslated mRNAs within stress granules under conditions of translational arrest [51].

Mutant KRAS also drives SG formation independently of eIF2α phosphorylation via the RAF–MEK–ERK (MAPK) pathway [52]. KRAS‐dependent dysregulation of prostaglandin metabolism, marked by elevated COX‐2 expression and suppressed 15‐hydroxyprostaglandin dehydrogenase (HPGD), leads to the accumulation of 15‐deoxy‐Δ^12,14^‐prostaglandin J2 (15‐d‐PGJ2). This metabolite covalently modifies and inhibits eIF4A, triggering SG assembly under oxidative, proteotoxic, UV, or chemotherapeutic stress. Notably, 15‐d‐PGJ2 secretion induces a paracrine stress‐response program, promoting SG formation in both KRAS‐mutant and neighboring KRAS‐wild‐type cells. Downstream recruitment of G3BP1, TIA1, and other SG nucleators stabilizes the ribonucleoprotein network, enhancing chemoprotection, stress resistance, and survival across the tumor microenvironment. Collectively, these findings establish KRAS as a central driver of SG assembly, linking oncogenic signaling to stress adaptation, apoptosis inhibition, and drug resistance in cancer.

## 4 Targeting SGs in KRAS‐Driven Cancer

Several small‐molecule inhibitors that directly disrupt SG assembly have been identified. Psammaplysin F, a naturally occurring compound isolated from marine sponges, prevents SG formation by inhibiting eIF2α phosphorylation, thereby interfering with canonical stress granule initiation [53]. Similarly, OSMI‐1, an inhibitor of O‐linked *N*‐acetylglucosamine transferase (OGT), reduces O‐GlcNAcylation of the SG nucleator UBAP2L, decreases its expression, and disrupts SG formation in response to sunitinib treatment [34]. In addition, two small‐molecule inhibitors, G3Ia and G3Ib, selectively block SG formation by targeting the nonstructural protein 3 (nsP3)‐binding pocket within the NTF2L domain of G3BP1/2 [54]. Since G3BP1 uses this domain to drive nucleation with SG components, these inhibitors bind the NTF2L domain and disrupt the multivalent protein–RNA network essential for SG condensation. Notably, KRAS‐mutant cells exhibit increased dependence on SG integrity because of chronic oncogenic and metabolic stress. It is reasonable to hypothesize that SG‐disrupting agents may sensitize KRAS‐driven tumors while sparing normal tissues.

In addition to targeting SG nucleators, recent work has highlighted KRAS‐specific vulnerabilities linked to stress‐inducible phase‐separating proteins. In the context of oncogenic KRAS^G12D^‐driven pancreatic tumorigenesis, nuclear protein 1 (NUPR1), an intrinsically disordered and stress‐inducible protein, was identified as a critical mediator of SG formation [26]. Genetic depletion of NUPR1 or its pharmacological inhibition by the small‐molecule compound ZZW‐115 prevents SG formation and selectively induces apoptosis in KRAS^G12D^‐expressing cells [26]. Treatment with ZZW‐115 reduces pancreatic intraepithelial neoplasia lesions in a KRAS^G12D^ pancreatitis/preneoplasia mouse model (Pdx1‐Cre; LSL‐KRAS^G12D^). These findings demonstrate that intrinsically disordered, phase‐separating proteins, which were considered undruggable, can be therapeutically targeted to destabilize the SG architecture in KRAS‐driven cancers.

Metabolic dependencies that sustain SG formation also represent actionable targets in KRAS‐mutant cancers. Stress granule assembly in mutant KRAS cells can be induced by 15d‐PGJ_2_ through the activation of NRF2 [52]. NRF2 is highly expressed in aggressive PDAC and is correlated with poor patient survival. Notably, glutamine starvation limits the ability of 15d‐PGJ_2_ to induce SGs [55]. Consistently, inhibition of glutaminase (e.g., with CB‐839) restored sensitivity to gemcitabine and suppressed tumor growth in vitro and in xenograft models, suggesting that targeting glutamine metabolism can overcome NRF2‐mediated, SG‐associated chemoresistance in KRAS‐driven PDAC. These observations align with the well‐established glutamine addiction of KRAS‐mutant tumors and position metabolic rewiring as a key upstream determinant of SG competence in KRAS‐driven cancer.

MG53 (TRIM72), a muscle‐derived E3 ubiquitin ligase of the TRIM family, interacts directly with the stress granule nucleator G3BP2 through its TRIM domain, forming a protein complex that alters G3BP2 localization [56]. This interaction promotes the nuclear translocation of G3BP2, reducing the pool of cytosolic G3BP2 available for multivalent interactions necessary for stress granule assembly. As a result, the formation of cytoplasmic stress granules under oxidative or chemotherapeutic stress is significantly diminished. By limiting SG assembly, MG53 impairs the protective role of stress granules in tumor cells. Consequently, NSCLC cells become more sensitive to chemotherapeutic agents, and tumor growth is suppressed.

A recently described axis connects vaccinia‐related kinase 2 (VRK2), G3BP1, and the E3 ubiquitin ligase RNF144A in the regulation of SG formation [57]. VRK2 directly phosphorylates G3BP1 at Ser149, a modification that suppresses SG formation. Under oxidative or chemotherapeutic stress, RNF144A is upregulated, promoting the proteasomal degradation of VRK2 and thereby relieving the inhibitory phosphorylation of G3BP1, which enables SG assembly. Depletion of VRK2 or overexpression of RNF144A enhances SG formation, whereas stabilization of VRK2 suppresses granule assembly and sensitizes cells to chemotherapy. These findings identify RNF144A‐mediated VRK2 loss as a key mechanism enabling G3BP1‐dependent SG formation and highlight this pathway as a potential therapeutic target to counteract SG‐driven chemoresistance.

Collectively, these studies support a model in which SGs function as adaptive stress‐response hubs, enabling KRAS‐mutant tumors to withstand oncogenic, metabolic, and therapeutic stress. Targeting SG nucleators, phase‐separating scaffolds, or the metabolic and posttranslational pathways that sustain SG assembly may therefore increase the efficacy of existing chemotherapeutic and targeted agents. Rational combination strategies integrating SG‐disrupting approaches with KRAS pathway inhibitors or metabolic therapies represent a promising avenue for overcoming resistance in KRAS‐driven malignancies.

## 5 Conclusion

Cancer cells exploit SGs as adaptive, cytoprotective assemblies that enable survival in hostile microenvironments characterized by metabolic, oxidative, and therapeutic stress. Oncogenic KRAS mutations have emerged as central drivers of aberrant SG formation and stabilization, thereby opening a novel therapeutic window for intervention. Despite recent advances in KRAS‐targeted therapies, such as KRAS inhibitors and the development of pan‐KRAS or mutant‐selective agents, significant challenges remain owing to pathway rewiring, rapid acquisition of drug resistance, and SG‐mediated prosurvival signaling that buffers stress and limits therapeutic efficacy. Although SG biology is still an emerging field and the full molecular complexity of SG assembly, regulation, and disassembly is not yet completely understood, accumulating evidence supports SGs as actionable vulnerabilities in cancer. Notably, SGs sequester proapoptotic factors and critical signaling proteins, and their disruption increases the sensitivity of cancer cells to apoptosis. Accordingly, targeting SGs has shown promise for overcoming drug resistance, which is a major obstacle in current cancer therapies. In addition, SGs have been implicated in maintaining cancer stemness programs, suggesting that their inhibition may not only enhance treatment responses but also improve long‐term patient outcomes.

The clinical relevance of SGs is further underscored by the association of core SG components with tumor progression, metastatic potential, and poor prognosis, positioning them as both therapeutic targets and candidate biomarkers. SGs are highly dynamic condensates composed of hundreds of proteins and RNAs; according to the Mammalian Stress Granule Proteome (MSGP) database (https://msgp.pt/, accessed November 18, 2025), at least 627 proteins have been identified in mammalian SGs. A deeper understanding of SG composition, dynamics, and regulatory networks may therefore aid in the discovery of biomarkers and the stratification of patients. Despite these advances, key questions remain unresolved, particularly regarding how and why oncogenic KRAS selectively exploits SGs to promote tumor aggressiveness, therapeutic resistance, and metabolic adaptation. While these mechanisms are still being elucidated, the accumulating mechanistic and therapeutic evidence presented in this review supports the concept that targeting SGs represents a viable and promising strategy in KRAS‐driven cancers. Continued investigations into SG regulation and KRAS‐SG crosstalk will be critical for the translation of this emerging biology into effective therapeutic interventions.

## Funding

Y.‐P.S. was supported by the National Science and Technology Council, Taiwan (MOST 111‐2320‐B‐039‐010, NSTC 112‐2320‐B‐039‐006, NSTC 113‐2320‐B‐039‐003, NSTC 112‐2622‐B‐039‐006, and NSTC 114‐2320‐B‐039‐057‐MY3), the National Health Research Institutes (EX112‐11219BI, EX113‐11219BI, EX114‐11219BI, and EX115‐11522BI), and the China Medical University (CMU112‐MF‐30, CMU113‐MF‐34, and CMU114‐MF‐03). This work was financially supported by the “Cancer Biology and Precision Therapeutics Center, China Medical University” from The Featured Areas Research Center Program within the framework of the Higher Education Sprout Project by the Ministry of Education (MOE) in Taiwan.

## Conflicts of Interest

The authors declare no conflicts of interest.

## Data Availability

Data sharing not applicable to this article as no datasets were generated or analyzed during the current study.
